# Influence of Process Energy on the Formation of Imperfections in Body-Centered Cubic Cells with Struts in the Vertical Orientation Produced by Laser Powder Bed Fusion from the Magnesium Alloy WE43

**DOI:** 10.3390/mi15020278

**Published:** 2024-02-15

**Authors:** Jan Jaroš, Ondřej Vaverka, Sascha Senck, Daniel Koutný

**Affiliations:** 1 Faculty of Mechanical Engineering, Institute of Machine and Industrial Design, Brno University of Technology, Technicka 2896/2, 616 69 Brno, Czech Republic; 2 Research Group Computed Tomography, University of Applied Sciences Upper Austria, Stelzhamerstraße 23, 4600 Wels, Austria

**Keywords:** laser powder bed fusion, magnesium alloy WE43, scanning strategy, lattice structure, relative density

## Abstract

The low specific density and good strength-to-weight ratio make magnesium alloys a promising material for lightweight applications. The combination of the properties of magnesium alloys and Additive Manufacturing by the Laser Powder Bed Fusion (LPBF) process enables the production of complex geometries such as lattice or bionic structures. Magnesium structures are intended to drastically reduce the weight of components and enable a reduction in fuel consumption, particularly in the aerospace and automotive industries. However, the LPBF processing of magnesium structures is a challenge. In order to produce high-quality structures, the process parameters must be developed in such a way that imperfections such as porosity, high surface roughness and dimensional inaccuracy are suppressed. In this study, the contour scanning strategy is used to produce vertical and inclined struts with diameters ranging from 0.5 to 3 mm. The combination of process parameters such as laser power, laser speed and overlap depend on the inclination and diameter of the strut. The process parameters with an area energy of 1.15–1.46 J/mm^2^ for struts with a diameter of 0.5 mm and an area energy of 1.62–3.69 J/mm^2^ for diameters of 1, 2 and 3 mm achieve a relative material density of 99.2 to 99.6%, measured on the metallographic sections. The results are verified by CT analyses of BCCZ cells, which achieve a relative material density of over 99.3%. The influence of the process parameters on the quality of struts is described and discussed.

## 1. Introduction

Laser powder bed fusion (LPBF) [1] is a method of additive processing of metal alloys, which is based on the principle of melting the powder particles layer by layer with a high-power laser. This enables the production of components with complex geometries from a variety of materials such as aluminum and titanium [2]. The development of materials for specific applications also takes place at LPBF, particularly in the field of alloys, which offer new possibilities [3,4].

One of these alloys is magnesium alloys, which have a low specific density and a good strength-to-weight ratio [5]. These properties are crucial for the use of magnesium alloys in the aerospace and automotive industries to reduce weight and improve fuel efficiency [6,7]. In addition, complex geometries such as lattice and bionic structures produced from magnesium alloys can enhance weight reduction [8,9]. Magnesium alloys such as WE43 are also biocompatible and biodegradable [10,11], making them suitable for biomedical applications such as implants due to their mechanical properties that are close to those of human bone [3,5,12].

In order to use magnesium alloy structures in industrial or medical applications, the imperfections associated with their manufacture must be minimized [13,14]. When manufacturing parts by LPBF, the thermal energy generated by the melting of the powder particles must be dissipated through the solid material in the preceding layers, as the surrounding powder has a lower thermal conductivity. Small volumes of material as lattice structures cannot dissipate the thermal energy quickly enough [15]. Therefore, the thermal energy is accumulated in the material [16] and causes the material to overheat, leading to the occurrence of imperfections such as porosity, high surface roughness and dimensional inaccuracy [17,18]. In order to minimize the occurrence of imperfections, process parameters must be used that lead to sufficient melting of the material and, at the same time, to sufficient dissipation of thermal energy [19,20].

The production of magnesium alloy lattice structures using LPBF technology is a particular challenge due to the narrow range of melting (650 °C) and boiling temperatures (1107 °C) [21]. The volume energy of process parameters of more than 214 J/mm^3^ (area energy of 8.56 J/mm^2^) leads to the vaporization of Mg in the magnesium alloy AZ61D [22]. The vaporization of alloying elements should also lead to a reduction in their mass ratio in alloys [23]. High energy input leads to an expansion of meltpool, which blasts away the surrounding powder. In contrast, cooling of the material reduces the volume and leads to the formation of pores due to the absence of powder particles [21]. Vapors also defocus the laser beam, which affects the quality of the manufactured parts [24]. Vaporization of the material can be prevented by using process parameters with lower thermal impact on the meltpool, which reduces the formation of vapors [22].

The processing of magnesium alloys should be divided into three stages depending on the energy of the process parameters [22,25]. As already mentioned, high-input energy leads to vaporization of the alloying elements. Low input energy may not always guarantee a sufficient temperature for melting the powder. Therefore, there is insufficient bonding between the molten material, resulting in low relative material density and poor mechanical properties [25]. Sufficient energy input leads to a reduction in the viscosity of the meltpool, which improves the effectiveness of the process [26,27]. The meltpool is stable, resulting in sufficient bonding between neighboring welds, which is necessary for the production of components with high relative material density and good mechanical properties. Hyer et al. [28] developed the process parameters for bulk samples of magnesium alloy WE43, leading to a high relative material density of 99.7%, i.e., laser power 200 W, scanning speed 1100 mm/s, hatch distance 0.13 mm, and layer thickness 0.04 mm.

For the production of small series components in the form of lattice structures, not only must process parameters be developed, but a specific scanning strategy should also be used [29]. The meander scanning strategy, which is mainly used for bulk samples, leads to the formation of porosity in the subsurface area due to the deceleration of the laser at the end of the trajectories [30]. The meander strategy also leads to high porosity in small-volume samples, as the trajectories of the laser cause the meltpool to overheat [20]. The contour scanning strategy is more efficient for lattice structures. Concentric welding trajectories allow for an effective energy distribution over the entire diameter of the strut. In addition, the contour scanning strategy has been successfully used to produce high-relative-material-density lattice structures [20].

Thus, the effective production of magnesium alloy lattice structures with minimal defects depends on the process parameters, scanning strategy, strut diameter and inclination. In this study, the production of vertical struts and inclined struts with an inclination of 35.26°, corresponding to Body-Centered Cubic (BCC) cells, is investigated. The most promising process parameters are evaluated on Body-Centered Cubic cells with struts in the vertical orientation (BCCZ), which are a combination of vertical and inclined struts. The magnesium alloy WE43 is used.

## 2. Materials and Methods

### 2.1. Laser Powder Bed Fusion

The samples are produced using the LPBF with the SLM 280HL machine (SLM Solutions Group AG, Lübeck, Germany). The machine is equipped with an ytterbium laser with a spot diameter of 82 μm and Gaussian distribution. A gas-atomized magnesium alloy WE43 (Luxfer MEL Technologies, Manchester, UK) with a particle size distribution of 28–60 μm with an average size of 39.8 μm is used. The chemical composition of the powder is listed in Table 1. Therefore, inert argon gas is used to prevent oxidation of the magnesium alloy during processing. The oxygen concentration is kept below a concentration of 0.1% during production. A laser power of 50–250 W and laser speed of 200–1200 mm/s are used for all experiments. The layer thickness is 50 μm and the temperature of the platform is set to 120 °C.

### 2.2. Contour Scanning Strategy

**Figure 1:**
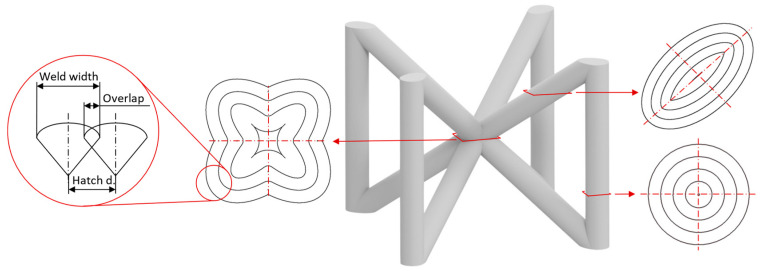
Parameters of contour scanning strategy and three different trajectories in BCCZ cell.

Based on previous research [20], the contour scanning strategy seems to be suitable for the production of lattice structures. The contour scanning strategy is formed by concentric welds that allow for better control of the input energy into the process, which is particularly important for low-volume samples (Figure 1). The most important parameters of the contour scanning strategy are the laser power and laser speed, which result in weld formations with an exact width. The next important parameter is the overlap of the neighboring welds, which is represented by the hatch distance. The optimal value of these parameters is the key to reducing imperfections in the material of lattice structures. Therefore, four experiments are conducted. The first experiment deals with the influence of the sample geometry and process parameters on the width of the weld. The second experiment deals with the hatch distance of hollow strut specimens formed by two welds. The output parameter of the experiment is the appropriate value of overlap for the production of specimens with high relative material density. Based on the parameters from the first two experiments, the contour scanning strategy is used in the third experiment to produce vertical and inclined struts with an inclination angle of 35.26% corresponding to BCC cell inclination. The last experiment is used to verify the best results of the third experiment. Therefore, the BCCZ cells representing both studied strut orientations are produced with different process parameters for vertical and inclined struts. In addition, three different laser trajectories of the contour strategy are identified in the BCCZ cells, which can influence the quality of the produced sample.

### 2.3. Weld Width

**Figure 2:**
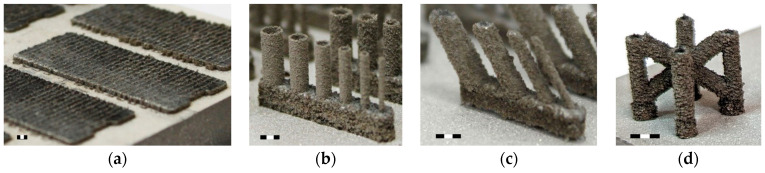
(**a**) Single-weld samples produced on the top of 1 mm thick WE43 material; (**b**) hollow struts; (**c**) inclined struts; (**d**) BCCZ cell (scale 3 mm).

The weld width depends on the process parameters, i.e., the laser power (LP) and the laser speed (LS) or their combination (Equation (1)), which is referred to as linear energy (LE). The linear energy of the process parameters in these experiments is in the range of 0.05–1.25 J/mm. Basically, the weld width is measured on single-track samples (Figure 2a), but the geometry of a single-track specimen does not correspond to the struts in the lattice structure. Therefore, samples with a thin-walled and hollow strut geometry (Figure 2b) are used to obtain more precise results, which are required for the contour scanning strategy. The weld width is included in the overlap of the neighboring welds (Equation (2)) as it changes with the geometry and diameter of the samples [20].

(1) $$LE=\frac{LP}{LS}\;(J/mm)$$

#### 2.3.1. Single-Weld Samples

**Figure 3:**
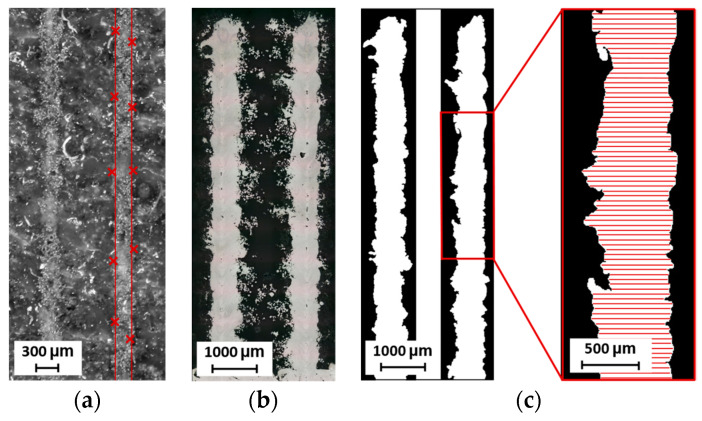
Measurement of sample width: (**a**) single weld sample; (**b**) metallographic section of hollow strut; (**c**) measurement of hollow strut width.

The width of the weld depends on the combination of laser power, laser speed and, in the case of single tracks, specifically on the substrate material. The weld width is also influenced by preheating the platform. Therefore, the single-track samples are produced on a 1 mm thick WE43 material (Figure 2a), which ensures adequate heat dissipation. The 1 mm thick material is fabricated with process parameters, i.e., laser power of 250 W, laser speed of 450 mm/s and hatch distance of 80 µm, based on the study [31]. The width of each weld is measured on the top images taken with an optical microscope (Olympus SZX7, Olympus, Tokyo, Japan). The width of single welds is the average value calculated from five measurements (Figure 3a).

#### 2.3.2. Thin Wall Samples

However, the geometry of the single-weld samples does not match the geometry of the struts in the lattice structure. Therefore, thin wall specimens are used to investigate the effects of the superposition of single welds. The upper weld partially melts the previous weld, increasing the width of the thin wall. The thin wall width is measured on the metallographic sections in the same way as the hollow strut specimens. The methodology is described below.

#### 2.3.3. Hollow Strut Samples

To obtain the most accurate weld width, the geometry of vertical hollow struts is used, created by a single weld with diameters from 0.5 to 3 mm with a step of 0.5 mm (Figure 2b). The width of hollow struts is influenced by heat dissipation, whereby the heat is mainly dissipated through the material of the specimen. However, due to the small volume of the specimen, the material is heated at higher temperatures, causing more powder particles to melt and increase in volume. Some of the heat is also dissipated through the surrounding powder particles and affects the surface of the samples, where the powder particles are partially melted and adhered.

The width of the thin walls and hollow struts is measured using metallographic sections that have been ground and polished (Figure 3b). The images of the cross-sections were taken with a digital microscope (Keyence VHX-6000, objective Z250R, zoom 250×, Keyence, Mechelen, Belgium). The width is the average value from both sides of the sample measured in each pixel of the image—approximately 8000 values are obtained (Figure 3c).

### 2.4. Overlap 

**Figure 4:**
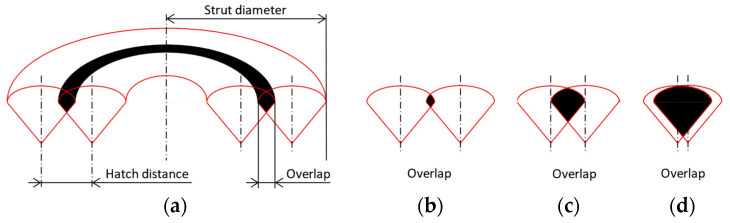
(**a**) Parameters in 2W hollow strut; visualization of overlap: (**b**) 10%; (**c**) 50%; (**d**) 90%.

The overlap of the neighboring weld together with the process parameters determines the energy density in the strut. The overlap (OL) depends on the weld width (WW) and hatch distance (HD) and represents the joint between two welds created by remelting the material (Equation (2), Figure 4). In the case of this study, the overlap is calculated from the width of the hollow struts. Therefore, it changes with the strut diameter. Lower overlap and laser power at a higher laser speed result in lower energy density. Low energy can result in areas of unmelted material being trapped in sharp-edged pores. High energy leads to overheated material, which supports the formation of spherical pores [16]. Therefore, the optimum value of overlap that suppresses pore formation and results in a sufficient bond between adjacent welds should be determined. For this purpose, the geometry of vertical hollow struts with two welds is used (Figure 4a). The hollow struts with two welds (2W) are produced with diameters from 0.5 to 3 mm with a step of 0.5 mm. Three values for laser power (100, 150 and 200 W) and laser speed (400, 600 and 800 mm/s) are chosen to cover the range of selected process parameters. The hatch distances are 0.06, 0.1 and 0.14 mm for each combination of laser power and laser speed, corresponding to an overlap of 20 to 80%. A total of 162 samples are produced. The optimum overlap is determined based on the porosity between two welds in the 2W hollow strut.

(2) $$OL=\left(\frac{WW-HD}{WW}\right)\cdot 100\;(\%)$$

### 2.5. Vertical and Inclined Struts 

The vertical and inclined (35.26°) struts (Figure 2c) are fabricated to determine the combinations of process parameters, i.e., laser power, laser speed and overlap, that result in high relative material density. Inclination of 35.26° corresponds to BCC cells, which are the worst case of inclination in the cubic crystal system and the most challenging to fabricate using LPBF technology. To categorize the influence of these parameters on the relative material density, the area energy (AE) is used (Equation (3)), where LP is the laser power, LS is the laser speed and HD is the hatch distance calculated according to Equation (2). Surface roughness and diameter deviation are also measured to capture the complex strut geometry. The experiment is designed by DOE with the following boundary conditions: laser power of 50–250 W; laser speed of 200–1000 mm/s, corresponding to a linear energy of 0.08–0.75 J/mm; and strut diameters of 0.5, 1, 2, and 3 mm. The overlap is between 45 and 85%, based on the results of the overlap experiment. These process parameters correspond to an area energy of 0.96–4.37 J/mm^2^. The DOE parameters for the vertical struts are 48 cube points, 12 center points in the cube, 48 axis points, and 12 center points in the axis. The inclined struts are designed with half of the DOE setup. A total of 120 vertical and 60 inclined struts are produced.

(3) $$AE=\frac{LP}{LS\cdot HD}({J/mm}^{2})$$

### 2.6. BCCZ Cells 

The BCCZ cells (Figure 2d) are produced to evaluate the best results of the vertical and inclined struts. The BCCZ cell is divided into vertical and inclined struts, which are produced with different process parameters depending on the result. The BCCZ cells are produced in 3 samples for each diameter (0.5, 1, 2 and 3 mm). CT analysis is used to evaluate the relative material density.

### 2.7. Relative Material Density

#### 2.7.1. Metallographic Sections

**Figure 5:**
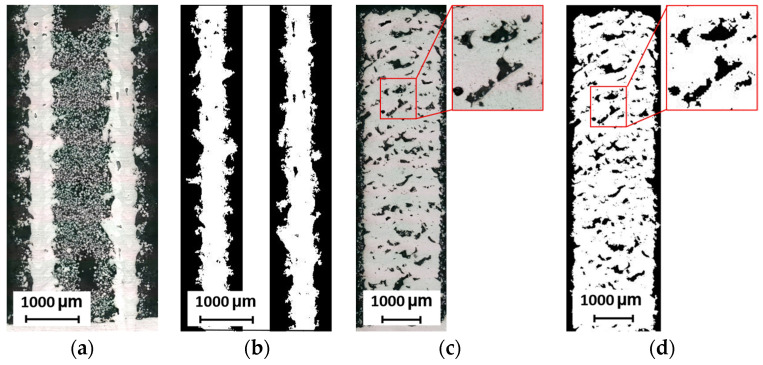
Relative material density measurement of: (**a**) 2W hollow strut; (**b**) 2W hollow strut monochrome image; (**c**) vertical strut with detail of pore; (**d**) vertical strut monochrome image.

Metallographic sections are used for two types of samples, i.e., 2W hollow struts and struts. The samples are metallographically processed and imaged with a Keyence digital microscope. The images are converted into binary monochrome images. Porosity is represented by the percentage of black areas in the sample section. For 2W hollow struts, the porosity is measured between the two adjacent welds according to the design of specimens (Figure 5a,b). The porosity of the struts is measured on metallographic sections (Figure 5c,d). In the contour scanning strategy, the center of the vertical and inclined struts as well as the lower part of the inclined struts is a critical point for porosity formation, as previous research has shown [19,20]. Therefore, the struts are polished towards the center and the relative material density values are used as a relative value for the comparison of the strut porosity.

#### 2.7.2. CT Analysis

Nanofocus X-ray computed tomography (nanoCT, GE phoenix|x-ray Nanotom 180 NF, Waygate technologies, Hürth, Germany) is used to analyze the relative material density in BCCZ cells. The following scanning parameters are used: an X-ray tube voltage of 130 kV, current of 80 µm, integration time of 600, average of 5 images, a total of 1500 projections, and a 0.1 mm copper filter to reduce beam-hardening artefacts. The final isovoxel resolution is 10 μm. Porosity analyses were carried out in VGStudioMax 2023.1 (Volume Graphics), using an ISO50 threshold and a minimum of eight voxels for a segmented pore, which is a typical threshold for the segmentation of pores [32].

### 2.8. Surface Roughness

**Figure 6:**
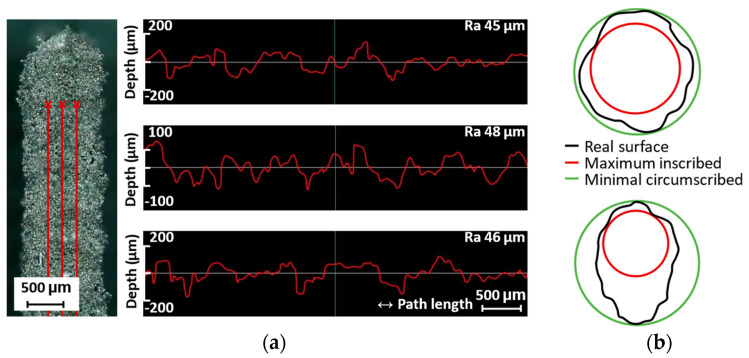
(**a**) Example of measurement of the surface roughness Ra on the side of the strut in three lines; (**b**) measurement of the dimensional deviation by fitting maximum inscribed and minimum circumscribed cylinders.

The surface roughness of the struts is mainly influenced by partially melted powder particles that adhere to the surface of the specimen. In the case of magnesium alloys, this effect is exacerbated by the low melting temperature. In the case of inclined struts, two effects occur. The first effect is the staircase effect, which is typical for additive manufacturing technologies and is mainly observed on the top side of the inclined samples. The second effect is related to heat dissipation, where heat is mainly transferred through the material towards the platform. The accumulation of heat causes the powder particles to melt and stick to the underside of the specimen. Therefore, the surface roughness Ra is measured on the side of the struts that is not affected by these two effects; moreover, it is also possible to compare the measured values between vertical and inclined struts, which are only affected by the process parameters. The surface roughness Ra is measured with a Keyence digital microscope. The surface of the struts is digitized, and the surface roughness Ra is measured in five lines parallel to the strut axis (Figure 6a). The final surface roughness Ra is the average of five measurements. The measured values are used to compare the influence of the process parameters and the orientation of the struts.

### 2.9. Diameter Deviation

The combinations of process parameters lead to different weld widths, which affect the dimensions of the manufactured samples. To evaluate this effect, the center of the outer laser track is set exactly to the diameter of the strut. Two parameters are measured, namely the maximum inscribed diameter and the minimum circumscribed diameter of the cylinders attached to the digitized struts (Figure 6b). The maximum inscribed cylinder represents the load-bearing diameter of the strut. The minimum circumscribed cylinder represents the amount of material melted on the surface of the struts. Figure 6b shows the differences between vertical and inclined struts, which differ in the lower part of the inclined struts. The diameters are measured with an optical 3D scanner (Atos Triple Scan III, GOM GmbH, Braunschweig, Germany). The scanned data are polygonised and the GOM Inspect software is used to fit the cylinders into the digitized surface of the struts using the best-fit method.

## 3. Results and Discussion

### 3.1. Effect of Specimen Geometry on Weld Width

**Figure 7:**
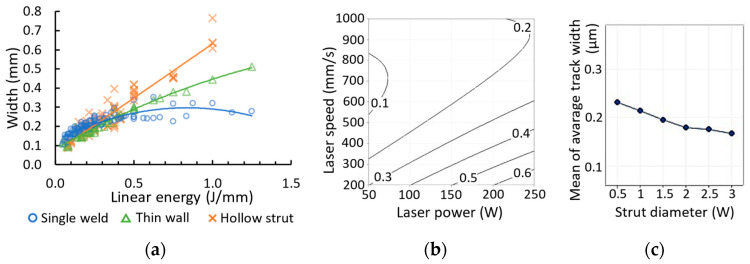
(**a**) Dependence of the width of single welds, thin walls, and hollow struts on linear energy; (**b**) width of hollow struts with a diameter of 1.5 mm based on the laser power and laser speed; (**c**) mean of average width dependent on strut diameter.

The width of the welds depends on the sample geometry, the process parameters and spot diameter size of the laser source. The width of the single welds, thin walls or hollow struts increases with the linear energy (Figure 7a). At a low linear energy of up to 0.4 J/mm, the widths show the same trend. At a linear energy of more than 0.4 J/mm, the geometry of the specimens leads to an increase in width for thin walls and hollow struts. For single welds, the width remains in a narrow interval between 0.23 and 0.32 mm. The width of all samples shows a dependence on the laser speed and laser power, which is illustrated by the results of the hollow struts in Figure 7b. The larger width is measured at a high laser power and low laser speed, corresponding to the influence of linear energy (Equation (1)). The width of the hollow strut (Figure 7c) also shows a slight dependence on the strut diameter, which is represented by the average values of the mean width.

In the contour scanning strategy, the weld width is an important parameter that determines the overlap between two concentric welds [29,33]. In many cases, the hatch distance is set as a fixed value and the influence of the process parameters is observed [13]. Therefore, the pores between two welds are formed as a function of the width of the weld. A small weld width leads to insufficient overlap and thus to a lack of fusion pores. A large weld width leads to a large overlap, which is typical for keyhole pores [34,35]. To reduce the formation of pores between two welds, the hatch distance must be different for different linear energies to ensure a constant overlap.

### 3.2. Overlap

**Figure 8:**
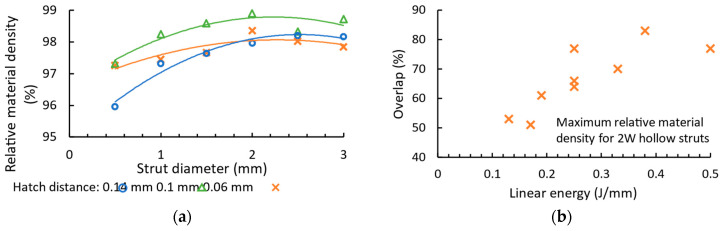

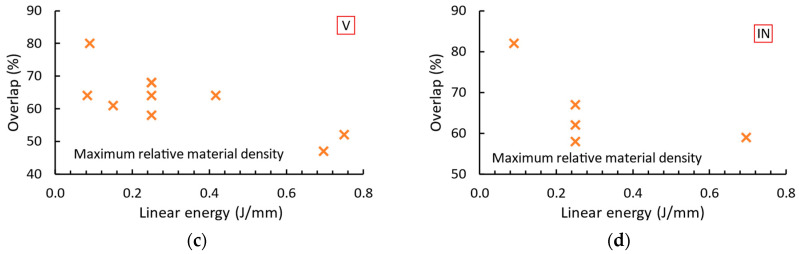
Dependence of (**a**) relative material density of 2W hollow strut on strut diameter for three batches of hatch distance produced with a laser power of 200W and laser speed of 800 mm/s; overlap on linear energy for the maximum measured relative material density of samples with diameter of 1 mm for (**b**) 2W hollow struts, (**c**) vertical struts, and (**d**) inclined struts.

For the 2W hollow struts, the hatch distance, which determines the overlap of welds (Equation (2)), shows a strong dependence on the strut diameter (Figure 8a) and linear energy (Figure 8b). The hatch distance in three batches for a linear energy of 0.25 J/mm shows a similar trend of relative material density in the range of 0.5–3 mm (Figure 8a). The maximum relative material density is measured at a hatch distance of 0.1 mm, which corresponds to an overlap of 60–68% according to the weld width and Equation (2). The overlap as a function of linear energy for the maximum relative material density for all combinations of process parameters and a diameter of 1 mm is shown in Figure 8b. The plotted values show that a high linear energy requires higher overlap values to achieve a high relative material density between two welds of 2W hollow struts. The overlap values are between 45 and 85% for all diameters; therefore, this range is used for the design of the strut experiment.

For the vertical and inclined struts with a diameter of 1 mm (Figure 8c,d), the dependence of the overlap for the maximum relative material density on the linear energy shows an increasing trend. An overlap of more than 80% leads to a relative material density of more than 99.3% for both vertical and inclined struts produced with a linear energy of less than 0.1 J/mm. An overlap of less than 60% results in a relative material density of over 98.4% at a linear energy of over 0.6 J/mm.

The overlap is the key parameter for the contour scanning strategy that influences the formation of pores between the welds [15,22]. The geometry of the samples influences the overlap required to obtain a high relative material density. The low-volume 2W hollow struts show an opposite trend than vertical and inclined struts (Figure 8b–d). A low linear energy of 2W hollow struts leads to a small weld width, which requires a smaller overlap. High energy leads to the opposite situation. This phenomenon can be related to heat dissipation when the material volume is able to dissipate a certain amount of heat [15]. 

In the case of the struts, the low linear energy requires a higher overlap in order to melt the material completely. For the high linear energy, a lower overlap is required. This is probably due to heat dissipation through the material of the entire strut [16]. The three situations occur for struts with a diameter of 2 mm (Table 2) based on the area considering the overlap (Equation (3)). The insufficient area energy of 1.34 J/mm^2^ for vertical strut (V) and 1.57 J/mm^2^ for inclined strut (IN) leads to irregular pores. The circular pores occur in the struts with an area energy of 3.99 (V) and 2.88 J/mm^2^ (IN). Area energies of 3.26 (V) and 2.46 J/mm^2^ (IN) lead to a high relative material density.

### 3.3. Relative Material Density

**Figure 9:**
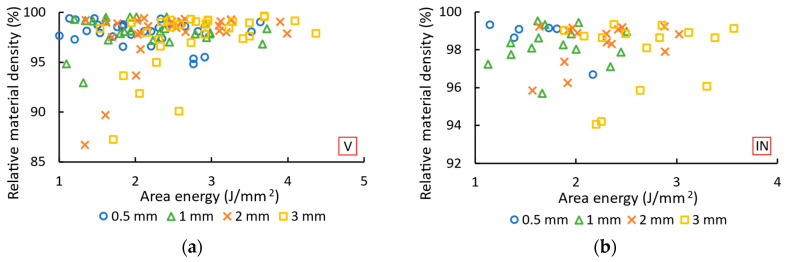
Relative material density depending on area energy for (**a**) vertical and (**b**) inclined struts with diameters of 0.5, 1, 2, and 3 mm.

The area energy (Equation (3)) is used to describe the effects of laser power, laser speed and overlap on the relative material density in the struts (Figure 9). The relative material density of the vertical struts (Figure 9a) increases with the area energy. An area energy of up to 2.6 J/mm^2^ leads to a relative material density of less than 95% for some of the vertical struts with a diameter of 1, 2 and 3 mm. In contrast, struts with a diameter of 0.5 mm are produced with a relative material density of over 97.6%. The area energy of over 2.6 J/mm^2^ leads to a relative material density of over 96.4% for diameters of 1, 2 and 3 mm. In the case of diameters of 0.5 mm, the relative material density is less than 95%. The relative material density of the inclined struts (Figure 9b) shows no clear dependency. The area energy of up to 2 J/mm^2^ appears to be promising for struts with a diameter of 0.5 mm. An area energy of more than 2 J/mm^2^ is more suitable for struts with a diameter of 2 mm.

The best results are shown in Table 3. The vertical and inclined struts achieved a relative material density of 99.2–99.6%. The optimal process parameters depend on the strut diameter. The inclination of the strut only affects the overlap. The vertical struts achieved a high relative material density with a higher area energy than the inclined struts (21–36% higher area energy). For the struts with a diameter of 0.5 mm, the area energy of 1.15–1.46 J/mm^2^ leads to a relative material density of over 99.3%. The struts with diameters of 1 mm, 2 mm and 3 mm achieve a relative material density of over 99.2% at an area energy of 1.62–3.69 J/mm^2^. The struts with diameters of 0.5 and 1 mm have small circular pores. The struts with diameters of 2 and 3 mm contain larger pores with an irregular shape.

The amount of area energy required to produce struts with a high relative material density depends on the diameter of the struts. The diameter of the struts influences the heat dissipation rate [15,16]. With a diameter of 3 mm, the heat dissipation rate is higher, which requires a higher area energy. For struts with diameters of 2 and 3 mm, the higher heat dissipation rate also leads to irregular pores in some parts of the strut. This type of pore is typical of insufficient energy [36].

For the production of BCCZ cells, the process parameters that lead to the best results in terms of relative material density are used. The CT scans are shown in Table 4. BCCZ cells with a diameter of 0.5 contain small spherical pores and an average relative material density of 99.96%. The BCCZ with a diameter of 1 mm achieves an average relative material density of 99.83% with a maximum pore volume of 0.011 mm^3^. BCCZ cells with diameters of 2 and 3 mm have irregular pores with a volume of more than 0.04 mm^3^.

**Figure 10:**
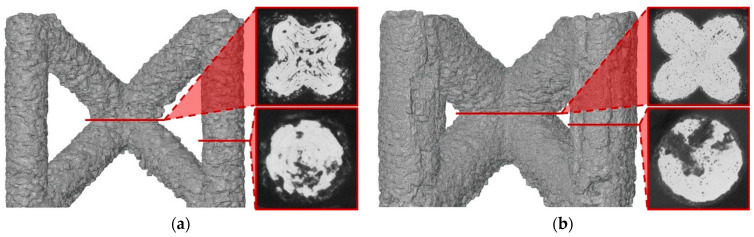
Polygonised surface of BCCZ cells and images of cross-sections of node and strut for diameters of (**a**,**b**).

In the BCCZ cells with a diameter of 2 mm, irregular pores appear between the welds (Figure 10a), which are typical for low energy. The low energy of the process parameters means that only part of the material is melted, leaving unmelted areas between the tracks in which unmelted particles are trapped [19]. The BCCZ cells (Figure 10b) with a diameter of 3 mm have irregular pores with a volume of more than 0.05 mm^3^ in the inclined struts, but the node of the cell is without large pores. This could be due to the laser trajectories, which are different for the cell node than for the struts (Figure 1). Due to the sharp edges in the center, the material is heated at a higher temperature, which supports the melting of the powder particles [35]. Vertical struts of 3 mm BCCZ cells showed deformations (holes) on the surface that go through the material of the struts and are not recognized as pores by the CT scan (Figure 10b).

**Figure 11:**
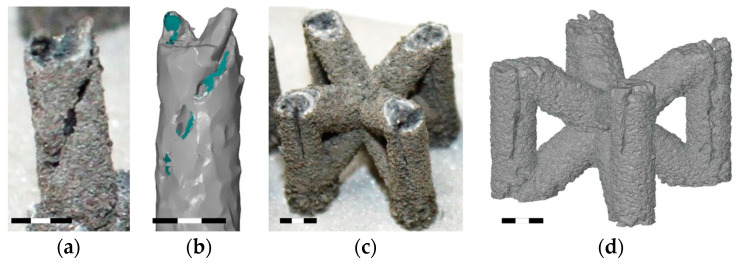
The holes on the surface of (**a**) vertical strut; (**b**) polygonised vertical strut; (**c**) BCCZ cell; (**d**) polygonised BCCZ cell (scale 3 mm).

These holes (Figure 11a–c) are related to the contour scanning strategy and are generated at the beginning on the outer laser trajectory [20]. These phenomena occur in the vertical struts with area energies of 2.3 J/mm^2^ for the vertical strut and 3.69 J/mm^2^ for the vertical strut of BCCZ cells, and a strut diameter of 3 mm. Therefore, the holes can be caused by LPBF melting of the powder particles in the contour strategy. The powder particles are drawn into the meltpool during melting [37], which is also favored by the low melting temperature of the magnesium alloy [21]. At the beginning of the outer laser trajectory, the meltpool draws all surrounded particles, resulting in the powder particles not forming a sufficient bond at the end of the laser trajectory. These holes can significantly affect the load-bearing part of the struts and serve as an initiator of crack during loading [38].

### 3.4. Surface Roughness

**Figure 12:**
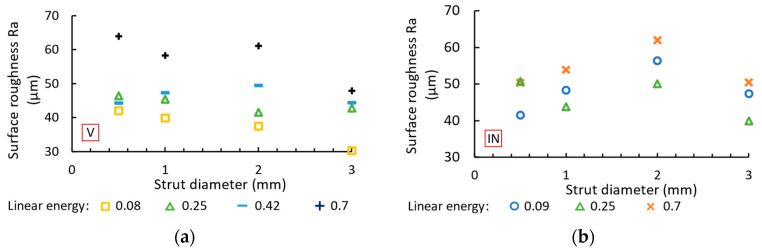
Surface roughness Ra for (**a**) vertical and (**b**) inclined struts.

The surface roughness Ra measured on the side of the struts is shown in Figure 12. Vertical (Figure 12a) and inclined (Figure 12b) struts are mainly influenced by the linear energy (laser power and laser speed), and the overlap parameters have no influence on the surface roughness. For vertical struts, the best results are measured at a linear energy of 0.08 J/mm, with a surface roughness Ra in the range of 30–42 µm. Inclined struts achieve an Ra of 42–50 µm at a linear energy of 0.09 J/mm (diameter of 0.5 mm) and a linear energy of 0.25 J/mm (diameter of 1, 2, 3 mm). A high linear energy (0.7 J/mm) leads to a surface roughness Ra of 48–64 µm for vertical struts and 51–62 µm for inclined struts.

The high surface roughness Ra is related to the low melting point of the magnesium alloy [21]. The heat from the meltpool is primarily dissipated via the material of the strut, but the small volume of the strut leads to overheating of the material. Therefore, part of the heat is dissipated via the surrounding powder particles, which have a lower heat dissipation rate [16]. The powder particles are partially melted and bonded to the surface, leading to an increase in the surface roughness Ra. 

### 3.5. Diameters of Vertical and Inclined Struts

**Figure 13:**
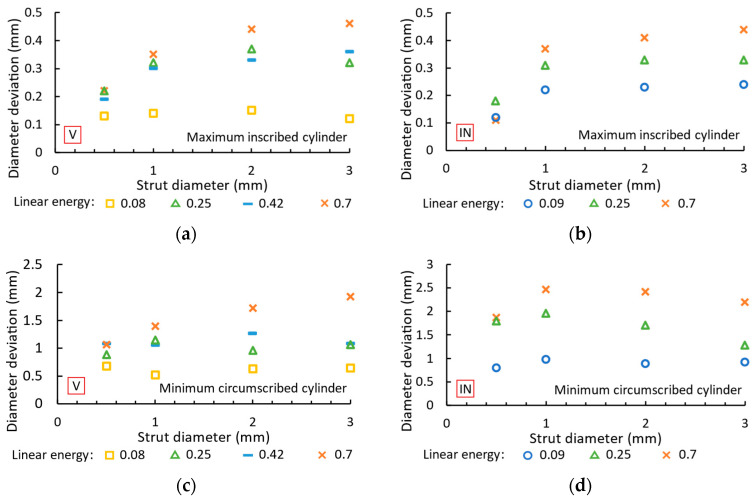
Diameter deviation of maximum inscribed cylinder and minimum circumscribed cylinder from required diameter for (**a**,**c**) vertical and (**b**,**d**) inclined struts.

The diameter deviations of the produced struts from the nominal diameters are listed in Figure 13, based on the maximum inscribed and minimum circumscribed cylinders. The maximum inscribed cylinder, which represents the load-bearing cross-section of the strut [39], reaches a larger diameter than required for both vertical (Figure 13a) and inclined (Figure 13b) struts. The deviation from the diameter increases with the linear energy and strut diameter. For vertical struts, a linear energy of 0.08 J/mm leads to a diameter deviation in the range of 0.12–0.15 mm. A linear energy of 0.7 J/mm leads to a diameter deviation of 0.22–0.46 mm. Inclined struts achieve a diameter deviation of 0.12–0.24 mm with a linear energy of 0.09 J/mm and 0.12–0.44 mm with a linear energy of 0.7 J/mm.

The minimum circumscribed cylinder means an increase in the strut diameter due to the high energy of the process parameters, which leads to the melting of more powder particles [20]. In addition, the partially melted powder particles are bound to the surface of the strut. With the inclined struts (Figure 13d), there is a lot of material on the underside as the heat is dissipated towards the platform. The deviation increases with the linear energy and strut diameter. Vertical struts (Figure 13c) show a minimum deviation of 0.52–0.68 mm at a linear energy of 0.08 J/mm and a maximum deviation of 1.07–1.93 mm at a linear energy of 0.7 J/mm. The inclined struts achieve a deviation of 0.8–1.79 mm (diameter of 0.5 mm) and 0.92–2.19 mm (diameter of 3 mm). The maximum diameter increases by 358% with a diameter of 0.5 mm and linear energy of 0.7 J/mm.

## 4. Conclusions

The study described the influence of the process parameters of contour strategy on the relative material density, surface roughness Ra and dimensional accuracy in the production of lattice structures from the magnesium alloy WE43. First, the experiments to determine the process parameters for the contour scanning strategy were evaluated. Then, the vertical and inclined struts were produced and the relative material density, surface roughness and dimensional deviation were analyzed. The best vertical and inclined struts with higher relative material density were used to fabricate BCCZ cells to verify the results. Based on the experiments, the following conclusions were drawn:

- The vertical and inclined struts achieved a relative material density of 99.3–99.6% with an area energy of the process parameters between 1.15 and 3.69 J/mm^2^ depending on the strut diameter and inclination.
- BCCZ cells with a diameter of 0.5 mm and 1 mm achieved a relative material density of 99.83–99.96% with small round pores. The BCCZ cells with a diameter of 2 mm and 3 mm achieved a relative material density of 99.32–99.72%.
- Holes were observed in the vertical struts of the 3 mm diameter BCCZ cells, which can be related to contour scanning strategy and large strut diameter.
- The overlap of the welds had a major influence on the porosity formation in the contour scanning strategy. The overlap value was determined based on the width of the weld of hollow strut specimens.
- The low melting point of the magnesium alloy WE43 led to an increase in the diameter of the struts. The load-bearing diameter deviated from the nominal diameter by 4–44% depending on the orientation and diameter of the strut.

## Figures and Tables

**Table 1:** Chemical composition of WE43 powder.

	Mg (wt %)	Y (wt %)	Zr (wt %)	Nd (wt %)	Si (wt %)	Cu (wt %)
WE43 powder	Bal.	3.96	0.56	2.30	<0.01	<0.01

**Table 2:** Comparison of the area energies of process parameters on vertical and inclined struts with a diameter of 2 mm.

LP (W)	250	50	150		221	79	79
LS (mm/s)	600	600	600		883	883	317
AE (J/mm^2^)	3.99	1.34	3.26		2.88	1.57	2.46
OL (%)	71	41	67		76	78	69
Vertical struts	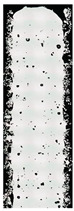	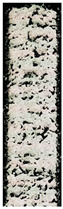	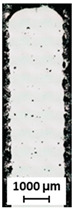	Inclined struts	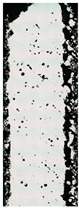	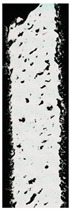	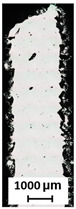
RD (%)	97.9	86.7	99.2		97.9	95.9	99.2

LP is laser power (W); LS is laser speed (mm/s); AE is area energy (J/mm^2^); OL is overlap (%); RD is relative material density (%); vertical and inclined orientation of struts.

**Table 3:** The best results obtained from the strut experiment.

LP (W)	79		79		79		150	
LS (mm/s)	883		317		317		600	
AE (J/mm^2^)	1.46	1.15	2.41	1.62	2.12	1.64	3.69	2.38
D (mm)	0.5		1		2		3	
OL (%)	75	75	64	58	54	54	69	64
Orient.	V	IN	V	IN	V	IN	V	IN
	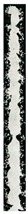	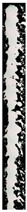	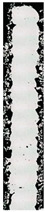	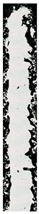	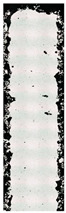	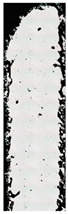	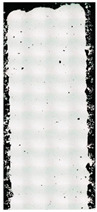	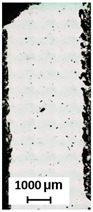
RD (%)	99.4	99.3	99.6	99.5	99.4	99.2	99.6	99.3

**Table 4:** CT scans of BCCZ cells.

D (mm)	0.5	1	2	3
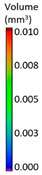	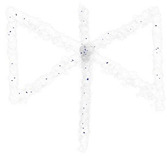	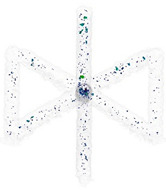	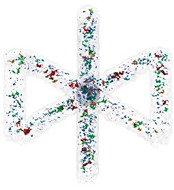	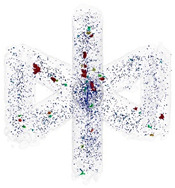
RD (%)	99.96	99.83	99.32	99.72

## Data Availability

Supporting data used for the publication are freely available at 10.5281/zenodo.10154211.
